# Risky sexual behaviors and associated factors among adult patients on antiretroviral treatment at Mankweng Hospital in Limpopo Province, South Africa

**DOI:** 10.3389/fpubh.2023.1245178

**Published:** 2023-10-12

**Authors:** Cairo B. Ntimana, Reneilwe G. Mashaba, Kagiso P. Seakamela, Tshifhiwa Netshapapame, Eric Maimela

**Affiliations:** ^1^DIMAMO Population Health Research Centre, University of Limpopo, Polokwane, South Africa; ^2^Department of Public Health, University of Limpopo, Polokwane, South Africa

**Keywords:** PLHIV, risky sexual behaviors, married, education, South Africa

## Abstract

**Background:**

Worldwide, it is estimated that 38 million people are HIV-positive and that over 36 million people have died from the virus. In South Africa, the prevalence of HIV was reported to be 20.6% with Limpopo Province having 17% HIV. Given the high rate of new HIV infection in Limpopo, there is therefore a need to assess factors promoting risky sexual behavior among people living with HIV in order to help design and develop behavioral interventions aimed at reducing risky behaviors among people living with HIV.

**Methods:**

This was a quantitative cross-sectional prospective study, conducted in Mankweng Hospital. The study consisted of 116 participants of which 40 were males and 76 were females aged 18 years and above. The participants were selected using purposive sampling. The data was analyzed using Statistical Package for Social Sciences version 27. A comparison of proportions was performed using Chi-Square. The association between risky sexual practice and sociodemographic factors was analyzed using multivariate logistic regression.

**Results:**

The proportion of risky sexual practices in the total population was 48.3%. Participants who were married, those aged 35–44, and those with tertiary qualifications were more likely to engage in risky sexual practices. Multivariate logistic regression showed widowed participants were less likely to practice risky sexual practices.

**Conclusion:**

The present study reported a high prevalence of risky sexual practices of 48.3%. Risky sexual behavior was determined by age, marital status, and level of education. The proportion of married participants was higher in risky sexual behavior. Based on the findings of the present study, it is recommended that targeted interventions and educational programs should be implemented to reduce risky sexual behavior among married individuals, individuals aged 35–44, and individuals with tertiary qualifications.

## 1. Introduction

HIV/AIDS is one of the leading causes of death worldwide. In 2020, 1.5 million individuals acquired AIDS globally, and approximately 680,000 people died from AIDS-related causes (1). By the end of 2014, it was estimated that 25.8 million persons in sub-Saharan Africa were HIV and AIDS-positive (2, 3). Despite the 90–90–90 treatment goal introduced by UNAIDS/WHO in 2014, which aimed that by 2020, 90% of all individuals should know their HIV status, 90% of PLHIV should be on treatment, and, 90% of those should have viral suppression (4), the new cases of HIV infection in sub-Saharan Africa seems to be increasing particularly in the southern regions (5, 6).

Reports on the sexual behaviors of individuals on HIV treatment in Africa have indicated that individuals on treatment (ART) are more likely to have unprotected sex compared to those who are not on treatment (7, 8). Although ART is increasing globally, the impact of ART on sexual risk behavior and transmission of HIV in Africa is still unknown (9). Although ART may minimize the risk of HIV transmission by persistent viral suppression, therapy may also serve as an excuse for unsafe sexual activities (10). There are several factors, which were reported as contributing factors to the spread of HIV among adults, such as domestic violence, having multiple partners, the practice of unprotected sex, homosexuality, and commercial sex (8, 11, 12).

Very little is known about the sexual behavior of PLHIV on ART in Limpopo, South Africa (13, 14). The incidence of HIV in South Africa is driven primarily by sexual contact between PLHIV and those who are uninfected, a growing number of South African PLHIV receiving ART were reported to continue engaging in unprotected sex (15). Risky sexual behavior among people receiving ART is a major public health concern, not only because of the risk of HIV transmission, but also the potential risk of transmission of resistant strains (16–19). People who default on treatment develop resistance to antiretroviral drugs leading to incomplete viral suppression, which may limit both the magnitude and the duration of the response to treatment (20). People who develop resistance as a result of default, when recommitting to the treatment, may engage in risky sexual behavior with the impression that the viral load is suppressed thus contributing to the incidence of HIV (21).

The prevalence of HIV in South Africa was reported to be 20.6%, with Limpopo Province having 17% HIV prevalence (22, 23). Given the high rate of new HIV infection in Limpopo (23–25), there is a need to assess factors promoting risky sexual behavior among PLHIV to help design and develop behavioral interventions aimed at HIV-positive individuals. As a result, the study sought to identify the factors that contribute to risky sexual behavior among PLHIV.

## 2. Methods and materials

### 2.1. Study design, and study setting

In this study, a quantitative study approach was utilized. A cross-sectional study design was implemented at Mankweng Hospital situated in the Capricorn District of the Limpopo Province in South Africa. The hospital is a government-funded tertiary institution which is a type of hospital that provides highly specialized medical care that involves complex procedures and treatment situated in the Mankweng township. Mankweng Hospital has a 550-bed capacity and has a Phela o Mphedise (*translation*; live and make me live) POP clinic. The POP Clinic provides services to an average of 2000 HIV/AIDS patients within Mankweng and the surrounding rural areas of Polokwane Local Municipality. The study was conducted from 2017 to 2018.

### 2.2. Sampling

The participants were selected using the purposive sampling method. Based on statistics, the Mankweng Hospital POP ART Clinic provided services to a total of 2000 PLWH aged ≥18 years receiving ART at the time of data collection. The total sample calculated using (*n* = 2000) was 322 ART patients. The sample size was calculated using Yamane’s population proportion approach formula in which a 5% sampling error provision was used (26). This was rounded up to 350 participants to allow for spoilt responses. As this study was voluntary, only 151 respondents took part in the study by completing the questionnaire. Of the 151 respondents, 35 of the questionnaires were spoilt and could not be used in the analysis making the total number of responses 116 participants.

### 2.3. Data collection

A 30-item questionnaire adapted from Purvis et al. (27) was given to patients, along with an introductory patient information form explaining the purpose of the research. The questionnaire covered 4 sections, namely (1) social-demographic data; (2) the sexual practices of the patients; (3) the health status of the patients; and (4) the use of alcohol and drugs. The participants who gave consent to participate in the research were offered a seat in a private room to complete the questionnaire, after signing a consent form. The data was collected from 1st January to the end of May 2018. Considering that Sepedi is the predominant language spoken among the study population, an option to choose between an English or Sepedi questionnaire was given to the participants. The participants’ information form and the consent form were read out to participants with no formal education to elicit their responses. Only those who gave consent to participate in the research were handed the questions and the options in the questionnaire were read out to them. The researchers recorded their responses on the survey form. To ensure the quality of data, data capturing and data cleaning were done by the researcher, which included removing outliers and participants with missing data.

### 2.4. Risky sexual behavior definition

In the present study risky sexual practice was defined as engaging in one of the following characteristics: unprotected oral sex, male-to-male sex, sex without the use of a condom or inconsistent use of a condom, and sexual intercourse under the influence of alcohol and drugs in the past 3 months (16, 18).

### 2.5. Data analysis

The data were analyzed using Statistical Package for Social Sciences (SPSS) version 27.0. Data were reported as frequencies and percentages. A comparison of proportions was performed using Chi-Square whilst a comparison of means was performed using an unpaired Student’s t-test. To determine the relationship between risky sexual behavior and associated factors we conducted a multivariate binary logistic regression. Risky sexual behavior was the exposure of interest. In the present study, the following variables were considered for the dependent variable, i.e., unprotected oral sex, male-to-male sex, sex without the use of a condom or inconsistent use of a condom, and sexual intercourse under the influence of alcohol and drugs. These variables were structured in a question format; the participants were asked if they have practiced any of the sexual practices listed in the following question; In the past 3 months, have you engaged in the following sexual practices, unprotected oral sex, male-to-male sex, sex without the use of a condom or inconsistent use of a condom, and sexual intercourse under the influence of alcohol and drugs in the past 3 months?. Risky sexual behavior was coded “1” if the participants answered yes to any of the sexual practices listed and coded “0” if they answered no to all the listed sexual practices. We adjusted for the main determinants of risky sexual practice which included sociodemographic (age, sex, educational status, and marital status). For all the inferential statistics, a *p*-value of less than 0.05 was considered statistically significant.

### 2.6. Ethical consideration

The study protocol was ethical approved by the Turfloop Research Ethics Committee (TREC) (TREC/320/2017:PG). The permission to conduct this study was granted by the Provincial Department of Health and the management of the Mankweng Hospital.

### 2.7. Rights of participants

The rights of the participants were not violated during this study and the principles of non-maleficence, human dignity, confidentiality, beneficence, and justice were maintained at all times.

### 2.8. Non-maleficence

During this research, none of the participants were harmed physically, verbally, or emotionally. A social worker and psychologist were made available, on referral, to counsel any participant who may have become distressed as a result of responding to the study questionnaire.

### 2.9. Human dignity

The researchers went through the informed consent form with the participants, making sure that they were aware of the study’s objectives and what was expected of them. The participants were made aware that they could opt out of participating in the research at any point, even after signing the consent form.

### 2.10. Confidentiality and privacy

No names of the participants were used in this research, instead, unique identifier codes were used to maintain the confidentiality of all questionnaires. The same level of confidentiality was applied when storing the data. All the data collected was kept on a laptop that was only accessed by the researcher. During data collection, participants were allowed to complete the questionnaires in a private space to maintain the privacy of all the participants.

### 2.11. Justice

All the participants in this study were treated equally and fairly and with a high degree of respect by the researcher. The researcher mentioned to the participants that participating in this research was voluntary and that there would be no material gain.

### 2.12. Beneficence

The results of this research will be presented to district health in Limpopo and to the national Department of Health. The outcome of the research will assist in collecting information that will help in the development of policies aimed at improving HIV and AIDS awareness.

## 3. Results

**Figure 1:**
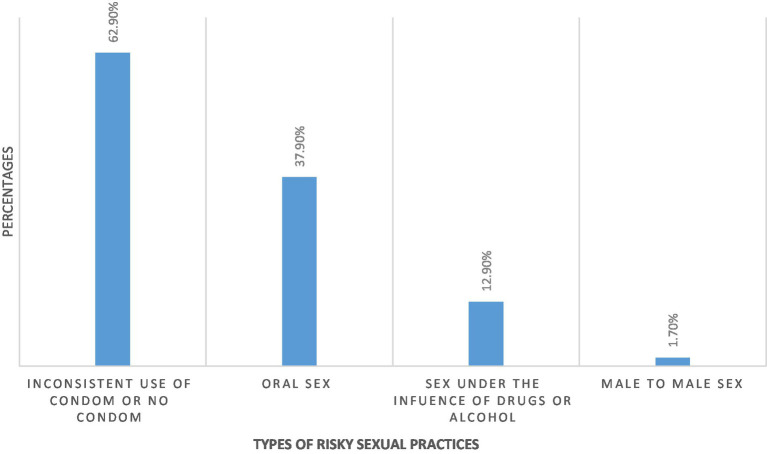
Shows the type of participants’ sexual risky practices.

All the participants who took part in the study were HIV positive. The majority of the participants were females. Most participants were of the age category of 35–44. In addition, the majority of the participants were single and had secondary school as their highest level of education (Figure 1 and Table 1).

**Table 1:** Sociodemographic characteristics of participants.

Variable	Frequency (n)	Percentage (%)
Gender		
Male	40	34.5
Female	76	65.5
Age		
18–24	2	1.7
25–34	12	10.3
35–44	51	44.0
45–54	31	26.7
>55	20	17.2
Marital status		
Single	56	48.3
Married	23	19.8
Divorced	23	19.8
Widowed	14	12.1
Highest level of education		
No formal education	18	15.5
Primary	10	8.6
Secondary	71	61.2
Tertiary	17	14.7

The prevalence of risky sexual practice in the present study was 48.3%. Amongst all participants who practiced risky behavior, 62.9% had sexual intercourse without a condom, 37.9% had oral sex, 12.9% had sexual intercourse under the influence of drugs and 1.7% had male-to-male sex.

There was no significant difference in gender (both males and females) between risky sexual behavior and non-risky sexual behavior. The proportion of participants aged 35–44 was significantly higher in participants with risky sexual behavior (56.8% vs. 36.1%, *p* = 0.048) as compared to those without risky sexual behavior. The proportion of participants aged 45–54 and those aged >55 was higher in participants without risky sexual behavior and compared to those with risky sexual behavior, respectively, (33% vs. 15.9%, *p* = 0.048), (19.4% vs. 13.6%, *p* = 0.048). There was no significant difference between risky sexual behavior and those without risky sexual behavior in single and divorced participants. The proportion of married participants was higher in risky sexual behavior (25.0% vs. 15.0%, *p* = 0.054) as compared to those without risky sexual behavior. The proportion of widowed participants was higher in non-risky sexual behavior (18.3% vs. 5.4%, *p* = 0.016) as compared to risky sexual behavior. There was no significant difference in sexual behavior between participants who had no formal education, primary and secondary school. However, risky sexual behavior (25.0% vs. 8.30%, *p* = 0.028) was significant in participants who had tertiary education (Table 2).

**Table 2:** Comparison of sociodemographic profile between risky sexual behavior and non-risky sexual behavior.

Variables		Risky sexual behavior	No risky sexual behavior	*p*-value
Gender	Male	17 (38.6)	23 (57.5)	0.547
	Female	27 (61.4)	49 (68.1)	
Age	18–24	2 (4.5)	0 (0.0)	0.048
	25–34	4 (9.1)	8 (11.1)	
	35–44	25 (56.8)	26 (36.1)	
	45–54	7 (15.9)	24 (33.3)	
	>55	6 (13.6)	14 (19.4)	
Marital status	Single	29 (51.8)	27 (45.0)	1.000
	Married	14 (25.0)	9 (15.0)	0.054
	Divorced	10 (17.9)	13 (21.7)	1.000
	Widowed	3 (5.4)	11 (18.3)	0.016
Level of education	No formal education	5 (11.4)	13 (18.1)	0.432
	Primary	3 (6.8)	7 (9.7)	0.740
	Secondary	25 (56.8)	46 (63.9)	0.556
	Tertiary	11 (25.0)	6 (8.3)	0.028

In multivariate regression, there was no relationship between risky sexual behavior with gender and age. Married participants were 2.60 (aOR = 2.60; 95%CI: 1.025–6.592) times more likely to practice risky sexual behavior (*p* = 0.044). Widowed participants were 0.106 (aOR = 0.106; 95%CI: 0.013–0.838) times less likely to practice risky sexual behavior (*p* = 0.033). There was no relationship between risky sexual behavior and level of education (i.e., No formal education, primary, and secondary). Participants with tertiary education were 3.669 (aOR = 3.669; 95%CI: 1.247–10.78) times more likely to practice risky sexual behavior (*p* = 0.018) (Table 3).

**Table 3:** Multivariate logistic regression of risky sexual behaviors and associated factors.

Variables		AOR (95%CI)	*p*-value
Gender	Female	1.341 (0.613;2.936)	0.462
	Male	1	1
Age			
18–24		0.254 (0.052;1.250)	0.092
25–34		0.880 (0.178;4.354)	0.8
35–44		1.883 (0.605;5.858)	0.274
44–54		0.643 (0.176;2.348)	0.504
>55		1	1
Marital status			
Single	Yes	0.965 (0.456;2.045)	0.926
	No	1	1
Married	Yes	2.60 (1.025;6.592)	0.044
	No	1	1
Divorced	Yes	1.065 (0.418;2.718)	0.895
	No	1	1
Widowed	Yes	0.106 (0.013;0.838)	0.033
	No	1	1
Level of education			
No formal education	Yes	0.582 (0.192;1.762)	0.338
	No	1	1
Primary	Yes	0.679 (0.166;2.777)	0.591
	No	1	1
Secondary	Yes	0.744 (0.346;1.600)	0.449
	No	1	1
Tertiary	Yes	3.667 (1.247;10.78)	0.018
	No	1	1

## 4. Discussion

The present study aimed to determine the prevalence of risky sexual behavior and its associated factors in PLHIV on ART, at Mankweng Hospital in Limpopo Province. In the present study, the prevalence of risky sexual behavior was 48.3%. These findings are congruent with the results of other studies conducted in Africa where the prevalence of risky sexual behavior was reported in Addis Ababa, Ethiopia, Johannesburg, South Africa, and Northwest Ethiopia at Gondar University Referral Hospital at 39.1, 30.4, and 38%, respectively (18, 28, 29). However, a study conducted in Zambia explicit a contrary results to this study in that it has reported a higher prevalence of risky sexual practice of 72.2% amongst participants aged 18–25 (30). The difference between the present study and the study conducted by Yang et al. could be the difference in age groups. In addition to oral sex, sex without the use of a condom, and sexual intercourse under the influence of alcohol, which were used to define risky sexual behavior in the present study, Yang et al. broadened the definition by including multiple sexual partners and casual sex with strangers thus likely contributing to the difference in prevalence.

In the present study, the proportion of the married category was significantly higher in risky sexual behavior as compared to non-risky sexual behavior. In addition, married participants were more likely to practice risky sexual behavior as compared to widowed. These findings concur with the results of a study conducted by Molla and Gelagay, which posits that married participants are more likely to engage in risky sexual behavior as compared to unmarried participants (29). Multivariate logistic regression showed that married participants were 2.60 times more likely to practice risky sexual behavior, a finding which is in agreement with research done by Tolmay et al. (31), who reported that married participants had the highest odds of inconsistence use of condoms as compared to those who are not married. The study found that low sexual decision-making capacity, relationship instability worry, and infidelity accusation fear were the main causes of inconsistent condom use in married women (31). Furthermore, Madiba and Ngwenya, cited that most men have indicated that they cannot pay lobola and still use a condom (32). In patriarchal societies, refusing unprotected sex or suggesting condom use is viewed as a threat to a husband’s authority or an indication of unfaithfulness, and may be confronted with physical abuse by the husband (31). In addition, a study by Tadesse and Gelagay (18), attributed the high prevalence of risky sexual behavior in married women to the need to have children, while some participants see no use of condoms since they are already infected with HIV.

The widowed category had a significantly higher proportion of non-risky sexual behavior as compared to risky sexual behavior. In multivariate binary logistic regression, widowed participants were 0.106 times less likely to practice risky sexual behavior. In agreement with the present study, reported widowed participants to be 0.30 times less likely to practice risky sexual behaviors study (33). The reason for widowed participants to practice less risky sexual behavior could be that, they are stigmatized in some areas which limits their social interactions (34).

There was no relationship between sexual behavior in participants with no formal education, primary, and secondary school. However, the proportion of risky sexual behavior was high in participants with tertiary education. In multivariate binary logistic regression, participants with tertiary education were 3.669 times more likely to practice risky sexual behavior. In agreement with the present study, Akinyi et al. reported a high proportion of inconsistent use of condoms in tertiary participants (35). There is still no clear explanation for why people with tertiary qualifications and living with HIV engage in risky sexual behaviors in rural South African populations. However, Ifeanyi Brian et al. (36) reported that 63.2% of tertiary students were engaging in risky sexual behavior for sexual experimentation and pleasure, oftentimes ignorant of the associated negative consequences.

### 4.1. Limitations of study

The study was conducted in rural Mankweng, which falls under the Capricorn District. The results, however, cannot be generalized to other rural communities as cultural and socio-demographic differences, depending on the area where research is conducted, and may influence sexual behaviors, sample size, the definition of risky sexual behavior, purposive sampling, and cross-sectional.

### 4.2. Recommendations

Based on the findings of the present study, it is recommended that targeted interventions and educational programs be implemented to address risky sexual behavior among married individuals, individuals aged 35–44, and individuals with tertiary qualifications. These interventions should focus on promoting safer sexual practices and increasing awareness about the potential risks associated with risky sexual behavior. Additionally, efforts should be made to improve sexual and reproductive health education, particularly among married individuals, to ensure they have the necessary knowledge and skills to make informed decisions about their sexual health.

## 5. Conclusion

The present study reported a high prevalence of risky sexual practice of 42.8%. Risky sexual behavior was determined by age, marital status, and the highest level of education. Married participants were more likely to practice risky sexual behavior. The present study found no significant difference in sexual behavior between participants who had no formal education, primary and secondary school. However, risky sexual behavior was found significant in participants who had tertiary education.

## Data availability statement

The raw data supporting the conclusions of this article will be made available by the authors, without undue reservation.

## Ethics statement

The studies involving humans were approved by the Turfloop Research Ethics Committee (TREC) (TREC/320/2017:PG). The studies were conducted in accordance with the local legislation and institutional requirements. The participants provided their written informed consent to participate in this study.

## Author contributions

CBN made substantial contributions to the conception, and design including analysis, and interpretation of data. RGM, KPS, TN, and EM made substantial contributions in methodology, interpretation of data, and reviewing and editing the manuscript. CBN, RGM, KPS, TN, and EM extensively reviewed the manuscript. All authors have read and agreed to the published version of the manuscript, read and approved the published version of the manuscript, collaboratively wrote the manuscript, did the design of the study including the data management and writing of the article, and involved in the study.
